# The Improvement of Air Quality and Associated Mortality during the COVID-19 Lockdown in One Megacity of China: An Empirical Strategy

**DOI:** 10.3390/ijerph18168702

**Published:** 2021-08-18

**Authors:** Zhihu Xu, Ru Cao, Xin Hu, Wenxing Han, Yuxin Wang, Jing Huang, Guoxing Li

**Affiliations:** Department of Occupational and Environmental Health Sciences, Peking University School of Public Health, 38 Xueyuan Road, Haidian District, Beijing 100191, China; zhihu_xu@sina.com (Z.X.); 13051555836@163.com (R.C.); xqxieqianqx@126.com (X.H.); hhwwxx499@163.com (W.H.); yuxin_wang@bjmu.edu.cn (Y.W.); jing_huang@bjmu.edu.cn (J.H.)

**Keywords:** lockdown policy, air pollution, difference-in-differences, health risk

## Abstract

Although the lockdown policy implemented during the COVID-19 pandemic indeed improved the air quality and reduced the related health risks, the real effects of the lockdown and its resulting health risks remain unclear considering the effects of unobserved confounders and the longstanding efforts of the government regarding air pollution. We compared air pollution between the lockdown period and the period before the lockdown using a difference-in-differences (DID) model and estimated the mortality burden caused by the number of deaths related to air pollution changes. The NO_2_ and CO concentrations during the lockdown period (17 days) declined by 8.94 μg/m^3^ (relative change: 16.94%; 95% CI: 3.71, 14.16) and 0.20 mg/m^3^ (relative change: 16.95%; 95% CI: 0.04, 0.35) on an average day, respectively, and O_3_ increased by 8.41 μg/m^3^ (relative change: 32.80%; 95% CI: 4.39, 12.43); no meaningful impacts of the lockdown policy on the PM_2.5_, PM_10_, SO_2_, or the AQI values were observed. Based on the three clearly changed air pollutants, the lockdown policy prevented 8.22 (95% CI: 3.97, 12.49) all-cause deaths. Our findings suggest that the overall excess deaths caused by air pollution during the lockdown period declined. It is beneficial for human health when strict control measures, such as upgrading industry structure and promoting green transportation, are taken to reduce emissions, especially in cities with serious air pollution in China, such as Shijiazhuang.

## 1. Introduction

The severe acute respiratory syndrome-coronavirus 2 (SARS-CoV-2) pandemic identified in 2019 (COVID-19) was reported to have caused 144,358,956 confirmed cases of coronavirus disease worldwide by 24 April 2021 [1]. The pandemic has changed almost every aspect of life worldwide. As asymptomatic infections play an important role in the spread of COVID-19 [2], many countries have adopted a series of restrictive measures to address the spread of the virus by implementing lockdown policies such as closing schools, workplaces, and public transport.

Some good news about the COVID-19 pandemic is that it has been reported that lockdown policies have positive implications for the environment. The air quality parameters of PM_2.5_, PM_10_, NO_x_, CO, and SO_2_ improved in many parts of the world during the lockdown period [3,4,5]. However, the concentrations of O_3_ were reported to have increased during the same period [3,5,6]. Lockdown policies have provided an opportunity to quantitatively evaluate the public health impacts resulting from resisting public transportation, closing factories, and balancing health and financial losses.

Although previous studies have shown improvements in air pollution during the lockdown period, these changes measured during the lockdown period could not represent the effect of the lockdown well because of confounders such as secular trends and weather influences [7,8]. Previous studies have assessed the health effects associated with air pollution during the Asia–Pacific Economic Cooperation (APEC) [9] and the 2015 China large-scale military parade periods [10], and one possible problem faced in these studies, as well as recent studies [11,12] during COVID, is a bias against the real effects of policies on air pollutants. The difference-in-differences (DID) model has been widely used to evaluate given interventions. A previous study applied a self-control DID method to evaluate the changes of air pollutants during the lockdown period [13]. This study tentatively used a DID analysis as an empirical strategy to explore the causal impacts of the lockdown policy on air pollutants; this analysis allowed us to control for omitted variables. We used the DID estimation technique to evaluate six air pollutants and the AQI during the lockdown period. To the best of our knowledge, this is the first study to use a case–control DID evaluation to calculate the health risks related to policy effects.

An outbreak of the COVID-19 pandemic occurred in Shijiazhuang, China, in January of 2021, and Shijiazhuang strictly implemented a city lockdown policy from 6 January [14] until 23 January [15]; this policy included closing workplaces, public transport and external transport with other cities, restricting gatherings, etc. This study assessed the changes in air pollution and related health risks that occurred during the lockdown period in Shijiazhuang based on a DID method.

## 2. Materials and Methods

### 2.1. Site Information of Air Pollutant Concentrations

Shijiazhuang and Baoding are relatively close in space. According to the statistical yearbooks of the two cities, the energy consumption patterns and high-emission industries shared similar levels between two cities, making these cities suitable for comparison. Based on the two reasons above, we finally chose Baoding as the control group in the DID model. There were eight air pollution monitoring sites in Shijiazhuang and six air pollution monitoring sites in Baoding; these sites were updated hourly.

### 2.2. Air Pollution and Weather Data

The city-level daily concentrations of six pollutants (SO_2_, NO_2_, CO, PM_10_, PM_2.5_, and O_3_) and the AQI for Shijiazhuang and Baoding of Hebei Province of China were collected from the daily air quality readings of the China National Environmental Monitoring Centre (CNEMC), obtained from local monitoring stations from December 20, 2020, to January 22, 2021. Since changes in pollutants are also often controlled by meteorological conditions [16], daily weather variables, including the daily average temperature, mean relative https://www.ogimet.com [humidity, and daily average wind speed, were also added to the model as covariables for analysis. Although precipitation removes air pollutants, no precipitation occurred in either city, and precipitation was not included in the analysis. All meteorological data were downloaded from the Synop stations and are available at accessd on 25 February 2021].

We compared the daily mean concentrations of six pollutants and the AQI values in different periods of lockdown (before and during) using the Wilcoxon test due to the non-normal distribution of pollutant data.

### 2.3. Baseline Number of Outcome Events

We assumed that the size of the baseline population changed minimally during the lockdown period. According to the Statistical Bulletin of National Economic and Social Development of Shijiazhuang [17], there were 10.39 million residents in Shijiazhuang at the time this study was performed. Based on an overall annual mortality rate of 5.2 individuals per thousand, the daily mortality count would be 148. According to a previous study [18], among the overall mortalities, the proportions of cardiovascular mortality and respiratory mortality were 43.74% and 12.32%, respectively, so it was estimated that there were approximately 65 and 18 deaths from cardiovascular and respiratory diseases, respectively, each day in Shijiazhuang.

### 2.4. DID Model

Although there have been many studies indicating the association of city lockdowns with improved air pollution, a key challenge is the endogeneity of city lockdowns originating from various confounding factors that potentially affect air pollution. One confounding factor is that the government has expended efforts to protect the environment, so the impact of the lockdown policy would be overestimated if we directly compared the two periods before and after the lockdown. To explore the realistic effect of the lockdown policy, we employed DID models. The DID models allowed us to control for various confounding factors and to identify the potential causal impacts of lockdown measures. Cities without lockdown policies, such as Baoding, can serve as counterfactuals and provide a reference level with which to study Shijiazhuang. We estimated the relative changes in air pollution levels between Shijiazhuang and Baoding using the following model (1):

(1) $$Y_{i,t}=\beta \times lockdown_{i,t}+\alpha \times Z_{i,t}+u_{i}+v_{t}+\varepsilon_{i,t}$$

where Y_i,t_ represents the level of air pollution in city i on date t; *lockdown*_i,t_ denotes whether a lockdown is enforced in city i on date t and takes the value of 1 if the city is locked down; Z_i,t_ are the control variables, including the daily average temperature, mean relative humidity, and daily average wind speed; *u*_i_ indicates city fixed effects; *v*_t_ indicates date fixed effects; *ε*_i,t_ is a disturbance term; *α* and *β* are the response parameters of the dependent variable.

We explored the effects of the lockdown policy using this DID model and evaluated the all-cause, cardiovascular, and respiratory disease deaths attributed to three distinct variations in pollutants.

### 2.5. Estimation of the Mortality Effects of a Unit Change in Daily Pollutant Concentrations

Previous studies have reported that the relationship between air pollutants and mortality is approximately linear without obvious threshold [19,20]. The exposure–response relationship is widely used to evaluate the health impact by many researchers [9,21]. In this study, the health impact assessment was conducted based on the exposure–response function and relevant baseline data about population size, baseline mortality, and changes in air pollution concentrations after calculating from the DID model. The numbers of avoided or increased deaths were attributed to the changes in pollutant concentrations based on the following formulas:

(2) $$\Delta_{\mathrm{number}\_of\_mortality}=baseline\_mortality\times \beta \times \Delta PC$$

(3) $$\Delta_{lower\_\lim it\_number\_of\_mortality}=baseline\_mortality\times \beta_{lower}\times \Delta PC$$

(4) $$\Delta_{upper\_\lim it\_number\_of\_mortality}=baseline\_mortality\times \beta_{upper}\times \Delta PC$$

where Δnumber_*of*_*mortality* is the estimated change in the number of deaths, *β* is the coefficient of the exposure–response function associated with daily pollutant concentration (per 1 μg/m^3^ change) and mortality, and Δ*PC* is the change in ambient pollutant concentrations.

To better evaluate the short-term health effects of air pollutants, we preferentially integrated the domestic studies as *β* [9,22,23,24] considering the susceptibility in different populations.

We obtained the all-cause mortality excess risk, cardiovascular mortality, and respiratory mortality from previously published time-series studies conducted in China on the three main kinds of pollutants (NO_2_, CO, O_3_); these pollutants were confirmed to significantly change through the DID process. According to a previous study [22], a 10 μg/m^3^ increase in the daily NO_2_ concentration in China would lead to a 0.65% (95% CI: 0.50%, 0.80%) increase in all-cause mortality, a 0.60% (95% CI: 0.41%, 0.79%) increase in cardiovascular mortality, and a 0.73% (95% CI: 0.46%, 1.00%) increase in respiratory mortality. Another study [18] of NO_2_ and linked mortality in China came up with some estimations and related health assessment could be found in supplement material. The estimates of the short-term associations among O_3_, CO, and all-cause, cardiovascular, and respiratory disease-related mortality are shown in Table 1.

### 2.6. Sensitivity Analysis

To test the validity of the DID estimate, we randomly moved up the time at which the lockdown policy was implemented in Shijiazhuang and checked if the coefficient estimate was significant.

All data analyses were conducted by R software (version 4.0.1, RStudio Team, Boston, MA, USA), the DID model was built by the “plm” package, and the level of significance was defined as *p* < 0.05 (two-tailed).

## 3. Results

### 3.1. Comparisons of Air Pollution Concentrations in Different Periods

After calculating the median and IQR concentrations of the AQI and pollutants, which are shown in Table 2, significant changes (*p* < 0.05) in NO_2_, O_3_, and SO_2_ were found in both Shijiazhuang and Baoding. The median concentration of NO_2_ was 21.90 (IQR = 12.99) μg/m^3^ during the lockdown days, while the median concentration of NO_2_ before the lockdown in Shijiazhuang was 55.01 (IQR = 26.48) μg/m^3^. Although NO_2_ decreased in both Shijiazhuang and Baoding during the lockdown period, it seems that the decline in Shijiazhuang was even greater than that in Baoding. SO_2_ showed the same behavior as NO_2_. O_3_ increased in both cities during the lockdown time. In contrast, CO in Shijiazhuang decreased significantly compared to that in Baoding.

### 3.2. DID Results

When controlling for weather variables (temperature, relative humidity, and wind speed), individual fixed effects, and time fixed effects, we estimated the relative changes in air pollutants and the AQI in Shijiazhuang relative to those in Baoding by fitting the DID model (Table 3). We found that the lockdown policy did improve the NO_2_ and CO situations, and the daily NO_2_ and CO declined by 8.94 μg/m^3^ (95% CI: 3.71, 14.16) and 0.2 mg/m^3^ (95% CI: 0.04, 0.35), respectively, when weather controls and fixed effects were included, indicating that those associated with the city lockdown were unlikely to be caused by weather or natural trends. The daily relative changes of NO_2_ and CO were 16.94% and 16.95%. The parameters of PM_10_, PM_2.5_, and the AQI were greater than zero but with no significance, indicating that no significant change occurred during the lockdown period. The daily concentration of O_3_ significantly increased by 8.41 μg/m^3^ (95% CI: 4.39, 12.43), with a 32.80% daily relative change.

**Figure 1:**
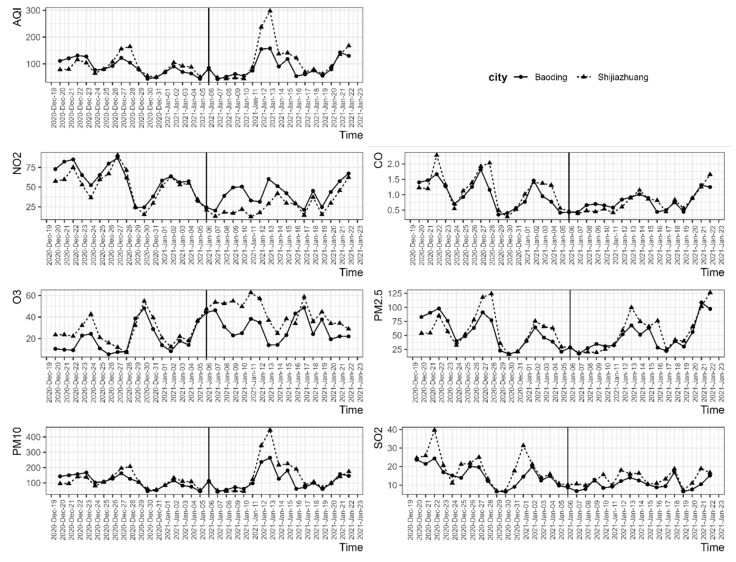
Time-series concentrations of air pollution in Shijiazhuang and Baoding. The solid black verticals are associated with the date of implementation of the lockdown policy.

We collected the daily average pollutant concentrations between Shijiazhuang and Baoding before and during the lockdown policy. Drawing time series of the pollutants between Shijiazhuang and Baoding allowed us to observe whether the parallel trend assumption was satisfied in the DID model. Figure 1 shows that no systematic difference was observed before the lockdown period between the two cities. In addition, the PM_2.5_, PM_10_, and AQI parameters displayed small declines within 3 days but were followed by a very large increase after the implementation of the lockdown policy.

When we randomly moved up the start time of the lockdown policy in Shijiazhuang, we found that the estimate of *β* gradually decreased and even became nonsignificant, reflecting that the lockdown policy indeed reduced the concentrations of related air pollutants, and our estimations were unlikely to be caused by other unobservable factors.

### 3.3. Evaluation of the Short-Term Health Benefits during the Lockdown Period

We evaluated the average daily excessive mortality of three clearly changed air pollutants after the DID analysis validation during the lockdown period. Among the estimates output by the DID model, the reductions in NO_2_ and CO could reduce the numbers of related deaths per day, while the effect of O_3_ was the opposite. The other pollutants did not change significantly during the lockdown period. According to Table 4, the number of all-cause excess deaths avoided by NO_2_ during the lockdown period (17 days) was 14.63 (95% CI: 11.25, 18.00), while the number of all-cause excess deaths that occurred due to O_3_ was 8.89 (95% CI: 6.77, 11.01). The numbers of deaths of cardiovascular diseases and respiratory diseases caused by the reduction in NO_2_ were also higher than the number of deaths caused by the increasing ozone concentration. Due to the lack of an exposure–response coefficient for all-cause and respiratory disease deaths due to CO, we only evaluated the number of deaths due to cardiovascular disease. Additionally, we combined all-cause results of the three pollutants through adding respiratory mortality from CO. Overall, we estimated that 8.22 (95% CI: 3.97, 12.49) all-cause deaths have been prevented during the lockdown period.

## 4. Discussion

This is the first study in China to evaluate the health benefits of the lockdown policy through the case–control DID method. We found that the lockdown policy reduced the concentrations of NO_2_ and CO but worsened the O_3_ situation, reducing all-cause deaths by 11.36 cases. The decline in SO_2_ was more likely linked to the lockdown policy, as described in previous studies [24,25,26]. However, no significant changes in SO_2_ and PM were observed, and the DID method helped us to decrease the bias and led to the separation of the causal effects of the lockdown policy.

The sources of air pollution are complicated and asymmetrical. As primary pollutants, CO and NO_2_ were directly influenced by reduced emissions during the lockdown. NO_2_ is created when air is heated and, on average, occurs due to transportation (15–20%) and fossil fuels in power plants (30–50%) [27], whereas residential (43.9%), transportation (29.4%), and industrial (25.1%) sources are the major sources of CO [28]. Given that residential heating was expected to increase during the lockdown period and given the insignificant changes in PM_2.5_ obtained herein (which is mainly generated by fossil fuel burning [29]), transportation played a crucial role in the relation between the air quality and lockdown policy. O_3_ is a secondary pollutant and is mainly generated by the photochemical reactions of nitrogen oxides and hydrocarbons from automobile exhaust under sunlight [29,30]. For O_3_, the seemingly abnormal increase may be derived from the decreased concentration of nitrogen oxides, which react with O_3_ [31]. The reductions in these primary pollutants slowed down their interactions with O_3_ and consequently elevated their levels; this effect was also observed in former studies [32,33].

PM represents the particle mass that enters the respiratory tract, and it includes both coarse and fine particles; the former is primarily produced by mechanical processes such as construction activities, road dust resuspension, and wind, whereas the latter originates mainly from combustion sources. We can see that PM_2.5_ and PM_10_ displayed short-term declines within three days after the lockdown policy was implemented, but the differences then decreased compared to Baoding, indicating that PM was not affected by the lockdown policy. A study in Austria showed a similar result: PM_10_ did not decline during the lockdown term, which might have been due to domestic heating [34]. It has been proven that coal combustion and PM are highly correlated [21]. Shijiazhuang strictly implemented the city lockdown policy from 6 January until 23 January. This period coincided with the heating period. According to the weather disaster warning information of the National Meteorological Center of CMA, a blue cold-wave warning was issued on 5 January [35]. Since the lockdown period in Shijiazhuang coincided with the heating period, fossil fuel heating may have caused a large amount of PM production. Research shows that during a cold wave in 2016, the cold wave increased the heating energy consumption by approximately 2.37% compared with 2014 [36]. Due to the lockdown policy, people may be more inclined to heating their home to a higher temperature than usual when they are at home [37]. All of the above factors may affect the PM level. Although the lockdown policy of the city reduced traffic flow, the PM level did not change significantly.

Using the DID method, we found that SO_2_ did not show a significant change due to the lockdown policy. A study in Wuhan, China, showed that during the lockdown period, atmospheric SO_2_ concentrations remained steady both annually and weekly [38]. This may be because industries are the most significant contributors, and smaller anthropogenic sources of SO_2_ emissions involve locomotives, ships, and vehicles with heavy equipment that burn fuel with a high sulfur content [39].

The overall impact of the COVID-19 epidemic is terrible, leading to death, disease, and economic loss. Many countries have had to implement lockdown policies [40]. In this study, we tentatively evaluated the health benefits of a lockdown policy, and the results indicate important implications for public health, even with the rise of O_3_. However, massive interventions were adopted during the lockdown period, and these interventions decreased air pollution but had high financial costs. Future research could explore these changes in air quality and the economic impacts of the lockdown and help leaders to implement rational policies in the future [41].

The reduction in traffic flow affected the level of air pollution, and we found that the NO_2_ concentration declined, while the AQI level had no significant change. Recent studies have shown that the key is to accelerate VOC emission reductions; this acceleration is expected to be achieved through the new emission standards for LDVs, and regional emission sources are expected to be simultaneously controlled to release the benefits of local traffic emission control, thereby improving the air quality [42]. The health benefits of the reduction in air pollution were proved significantly in a nationwide study [43]. A recent study also showed that the lockdown policy provided a good opportunity to test measures such as telecommuting, telemedicine, online business, and education [44]. These measures reduce people’s travel and contact and have beneficial impacts associated with the improvement of air pollution; thus, these measures may promote the development of smart cities.

We used a relative case–control DID method to study the real effect of the COVID-19 lockdown policy on air pollution levels and the associated mortality burden in the city of Shijiazhuang. However, some limitations still exist. First, because Shijiazhuang was the only city with a COVID-19 outbreak during the study period, we could only include one city as an intervention group for this research, causing the external validity of the results to be limited to some extent. Second, we did not consider changes in residents’ behaviors caused by the lockdown policy. The regulations implemented during the lockdown policy limited the behaviors of individuals, such as going outside. At the same time, the fact that we applied the annual mortality rate to infer the daily mortality is not very appropriate for our study period because the daily mortality is usually higher than the annual ones. Thus, bias in our results may exist in some way when estimating the impact of the health benefits of lockdown policies. Multicity studies and population activity models with empirical evaluations of policy effects are needed in the future. Third, although this study controlled for confounders that do not vary with time and are individual (city geography, city industrial capacity, etc.), it may not control all potential confounders that could lead to uncertainty. 

## 5. Conclusions

Our study found that the average daily concentrations of NO_2_ and CO were reduced and the concentration of O_3_ was elevated under the impact of the lockdown policy. The excess number of deaths per day caused by these three significantly changing pollutants decreased. Lockdown policies, such as restrictions on public health and gatherings, are effective in alleviating air pollution and reducing health risks.

## Figures and Tables

**Table 1:** The association between daily NO_2_, CO, and O_3_ concentrations and mortality obtained from previous studies in China.

	Study	Study Period	Daily Increment	Death Cause	*β*^a^ (95% CI)
NO_2_^b^	Li, 2021 [23]	2013–2017	10 ug/m^3^	All-cause mortality	0.65% (0.50%, 0.80%)
				CVD mortality	0.60% (0.41%, 0.79%)
				Respiratory mortality	0.73% (0.46%, 1.00%)
CO	Liu, 2018 [18]	2013–2015	1 mg/m^3^	All-cause mortality	NA
				CVD mortality	1.12% (0.42%, 1.83%)
				Respiratory mortality	NA
O_3_	Yan, 2013 [24]	meta-analysis	10 ug/m^3^	All-cause mortality	0.42% (0.32%, 0.52%)
				CVD mortality	0.44% (0.17%, 0.70%)
				Respiratory mortality	0.50% (0.22%, 0.77%)

^a^ *β* and its 95% CI refer to coefficients and corresponding 95% CI for the association between daily increment of air pollutants and mortality. ^b^ Chen et al. [24] reported association between NO_2_ and related mortality in 2018 as well; related health impact assessment can be found in Supplementary Material Table S1.

**Table 2:** Summary statistics of the AQI and pollutants from 20 December 2020 to 22 January 2021.

	20 December 2020–5 January 2021 ^a^		6 January 2021–22 January 2021 ^a^			
Shijiazhuang	Median	IQR	Median	IQR	*W* ^b^	*p*-Value
AQI	81.06	35.01	87.47	75.33	139	0.865
CO	1.23	0.83	0.60	0.42	216	0.013
NO_2_	55.01	26.48	21.90	12.99	246	0.000
O_3_	22.26	14.14	44.78	19.63	39	0.000
PM_10_	105.26	53.32	108.40	118.82	132	0.683
PM_2.5_	54.00	39.85	41.78	49.82	161	0.586
SO_2_	20.66	11.29	12.19	5.77	212	0.020
Baoding						
AQI	79.60	42.62	75.14	62.72	152	0.812
CO	0.95	0.70	0.70	0.39	194	0.092
NO_2_	61.64	20.40	42.33	21.45	224	0.005
O_3_	13.90	15.15	25.04	16.27	65	0.005
PM_10_	107.93	60.26	97.68	83.85	154	0.760
PM_2.5_	48.50	38.28	34.65	27.87	172	0.357
SO_2_	14.87	7.73	9.42	4.05	219	0.009

^a^ The dates of the two phases in study, both are 17 days. ^b^ *W* means Wilcoxon statistic.

**Table 3:** The effects of lockdown on air pollutants and the AQI ^a^.

	AQI	CO	NO_2_	O_3_	PM_10_	PM_2.5_	SO_2_
β_lockdown_^b^	17.36	−0.20 **	−8.94 ***	8.41 ***	27.24	0.59	−0.73
Standard error	11.95	0.08	2.67	2.05	16.58	5.54	1.44
*p*	0.157	0.018	0.002	0.000	0.111	0.916	0.617
R^2^	0.876	0.946	0.969	0.962	0.900	0.935	0.904
Observations	68	68	68	68	68	68	68

** *p* < 0.05; *** *p* < 0.001. ^a^ The model has controlled weather variables (daily temperature, relative humidity, and wind speed), city fixed effect, and time fixed effect. ^b^ Daily concentration change on average estimated by DID method during the lockdown period.

**Table 4:** Number of deaths due to air pollutants change during the lockdown.

	*β_lockdown_*	Cause of Death	Daily Deaths	95%CI	Total Deaths (17 days)	95% CI
NO_2_	−8.94	All-cause deaths	−0.86 ^a^	−(0.66,1.06)	−14.63	−(11.25,18.00)
RC ^c^	16.94%	Cardiovascular disease deaths	−0.35	−(0.24,0.46)	−5.93	−(4.05,7.80)
		Respiratory disease deaths	−0.12	−(0.07,0.16)	−2.00	−(1.26,2.74)
CO	−0.20	All-cause deaths				
RC	16.95%	Cardiovascular disease deaths	−0.15	−(0.05,0.24)	−2.48	−(0.93,4.04)
		Respiratory disease deaths				
O_3_	8.41	All-cause deaths	+0.52 ^b^	+(0.40,0.65)	+8.89	+(6.77,11.01)
RC	32.80%	Cardiovascular disease deaths	+0.24	+(0.09,0.38)	+4.09	+(1.58,6.51)
		Respiratory disease deaths	+0.08	+(0.03,0.12)	+1.29	+(0.57,1.98)
Summary		All-cause deaths	−0.48	−(0.23,0.73)	−8.22	−(3.97,12.49)

^a^ The “−” indicates the number of deaths decreased due to reduced pollutant. ^b^ The ”+” indicates the number of deaths increased due to increased pollutant. ^c^ RC: relative changes of air pollutants when comparing to the non-lockdown period.

## Data Availability

The weather data is gained from China National Environmental Monitoring Centre (CNEMC) and the meteorological conditions could be found online at https://www.ogimet.com accessed on 10 June 2021.

## Supplementary Materials

The following are available online at https://www.mdpi.com/article/10.3390/ijerph18168702/s1, Table S1. Number of deaths due to NO_2_ change during the lockdown based two references.

## References

[1] World Health Organzation Coronavirus Disease (COVID-19) Pandemic. https://www.who.int/emergencies/diseases/novel-coronavirus-2019.

[2] Gandhi M., Yokoe D.S., Havlir D.V. (2020). Asymptomatic Transmission, the Achilles’ Heel of Current Strategies to Control COVID-19. N. Engl. J. Med..

[3] Naqvi H.R., Mutreja G., Shakeel A., Siddiqui M.A. (2021). Spatio-temporal analysis of air quality and its relationship with major COVID-19 hotspot places in India. Remote Sens. Appl..

[4] Lau H., Khosrawipour V., Kocbach P., Mikolajczyk A., Schubert J., Bania J., Khosrawipour T. (2020). The positive impact of lockdown in Wuhan on containing the COVID-19 outbreak in China. J. Travel Med..

[5] Bekbulat B., Apte J.S., Millet D.B., Robinson A.L., Wells K.C., Presto A.A., Marshall J.D. (2021). Changes in criteria air pollution levels in the US before, during, and after COVID-19 stay-at-home orders: Evidence from regulatory monitors. Sci. Total Environ..

[6] Achebak H., Petetin H., Quijal-Zamorano M., Bowdalo D., Pérez García-Pando C., Ballester J. (2021). Trade-offs between short-term mortality attributable to NO2 and O3 changes during the COVID-19 lockdown across major Spanish cities. Environ. Pollut..

[7] Lin J.T., Liu M.Y., Xin J.Y., Boersma K.F., Spurr R., Martin R., Zhang Q. (2015). Influence of aerosols and surface reflectance on satellite NO_2_ retrieval: Seasonal and spatial characteristics and implications for NO*x* emission constraints. Atmos. Chem. Phys..

[8] Zhang H., Lin Y., Wei S., Loo B.P.Y., Lai P.C., Lam Y.F., Wan L., Li Y. (2021). Global association between satellite-derived nitrogen dioxide (NO(2)) and lockdown policies under the COVID-19 pandemic. Sci. Total Environ..

[9] Liu Q., Huang J., Guo B., Guo X. (2017). Efficiency of Emission Control Measures on Particulate Matter-Related Health Impacts and Economic Cost during the 2014 Asia-Pacific Economic Cooperation Meeting in Beijing. Int. J. Environ. Res. Public Health.

[10] Lin H., Liu T., Fang F., Xiao J., Zeng W., Li X., Guo L., Tian L., Schootman M., Stamatakis K.A. (2017). Mortality benefits of vigorous air quality improvement interventions during the periods of APEC Blue and Parade Blue in Beijing, China. Environ. Pollut..

[11] Giani P., Castruccio S., Anav A., Howard D., Hu W., Crippa P. (2020). Short-term and long-term health impacts of air pollution reductions from COVID-19 lockdowns in China and Europe: A modelling study. Lancet Planet. Health.

[12] Venter Z.S., Aunan K., Chowdhury S., Lelieveld J. (2021). Air pollution declines during COVID-19 lockdowns mitigate the global health burden. Environ. Res..

[13] Chen K., Wang M., Huang C., Kinney P.L., Anastas P.T. (2020). Air pollution reduction and mortality benefit during the COVID-19 outbreak in China. Lancet Planet. Health.

[14] Health Commission of Hebei Province Shijiazhuang Released 10 Measures for Community Prevention and Control: Non-Residents Strictly Control Entry and Exit. http://www.hebwsjs.gov.cn/html/sxdt/20210106/375242.html.

[15] Health Commission of Hebei Province (2021). Starting Today. Shijiazhuang Implements District and Hierarchical Control. http://www.hebwsjs.gov.cn/html/sxdt/20210123/375841.html.

[16] http://tjj.sjz.gov.cn/col/1584345215439/2020/04/13/1587111783229.html.

[17] Ma W., Zeng W., Zhou M., Wang L., Rutherford S., Lin H., Liu T., Zhang Y., Xiao J., Zhang Y. (2015). The short-term effect of heat waves on mortality and its modifiers in China: An analysis from 66 communities. Environ. Int..

[18] Chen R., Yin P., Meng X., Wang L., Liu C., Niu Y., Lin Z., Liu Y., Liu J., Qi J. (2018). Associations Between Ambient Nitrogen Dioxide and Daily Cause-specific Mortality: Evidence from 272 Chinese Cities. Epidemiology.

[19] Chen K., Breitner S., Wolf K., Stafoggia M., Sera F., Vicedo-Cabrera A.M., Guo Y., Tong S., Lavigne E., Matus P. (2021). Ambient carbon monoxide and daily mortality: A global time-series study in 337 cities. Lancet Planet Health.

[20] Wang X., Mauzerall D.L. (2006). Evaluating impacts of air pollution in China on public health: Implications for future air pollution and energy policies. Atmos. Environ..

[21] Li J., Zhang X., Li G., Wang L., Yin P., Zhou M. (2021). Short-term effects of ambient nitrogen dioxide on years of life lost in 48 major Chinese cities, 2013–2017. Chemosphere.

[22] Liu C., Yin P., Chen R., Meng X., Wang L., Niu Y., Lin Z., Liu Y., Liu J., Qi J. (2018). Ambient carbon monoxide and cardiovascular mortality: A nationwide time-series analysis in 272 cities in China. Lancet Planet. Health.

[23] Yan M., Liu Z., Liu X., Duan H., Li T. (2013). Meta-analysis of the Chinese studies of the association between ambient ozone and mortality. Chemosphere.

[24] Liu F., Wang M., Zheng M. (2021). Effects of COVID-19 lockdown on global air quality and health. Sci. Total Environ..

[25] Chen G., Tao J., Wang J., Dong M., Li X., Sun X., Cheng S., Fan J., Ye Y., Xiao J. (2021). Reduction of air pollutants and associated mortality during and after the COVID-19 lockdown in China: Impacts and implications. Environ. Res..

[26] Bao R., Zhang A. (2020). Does lockdown reduce air pollution? Evidence from 44 cities in northern China. Sci. Total Environ..

[27] Rohde R.A., Muller R.A. (2015). Air Pollution in China: Mapping of Concentrations and Sources. PLoS ONE.

[28] Liu X.Y., He K.B., Zhang Q., Lu Z.F., Wang S.W., Zhang Y.X., Streets D.G. (2019). Analysis of the origins of black carbon and carbon monoxide transported to Beijing, Tianjin, and Hebei in China. Sci. Total Environ..

[29] Scovronick N., Wilkinson P. (2014). Health impacts of liquid biofuel production and use: A review. Glob. Environ. Chang..

[30] Zeng P., Lyu X.P., Guo H., Cheng H.R., Jiang F., Pan W.Z., Wang Z.W., Liang S.W., Hu Y.Q. (2018). Causes of ozone pollution in summer in Wuhan, Central China. Environ. Pollut.

[31] Marr L.C., Harley R.A. (2002). Spectral analysis of weekday–weekend differences in ambient ozone, nitrogen oxide, and non-methane hydrocarbon time series in California. Atmos. Environ..

[32] Sicard P., De Marco A., Agathokleous E., Feng Z., Xu X., Paoletti E., Rodriguez J.J.D., Calatayud V. (2020). Amplified ozone pollution in cities during the COVID-19 lockdown. Sci. Total Environ..

[33] Tobías A., Carnerero C., Reche C., Massagué J., Via M., Minguillón M.C., Alastuey A., Querol X. (2020). Changes in air quality during the lockdown in Barcelona (Spain) one month into the SARS-CoV-2 epidemic. Sci. Total Environ..

[34] Lovrić M., Pavlović K., Vuković M., Grange S.K., Haberl M., Kern R. (2021). Understanding the true effects of the COVID-19 lockdown on air pollution by means of machine learning. Environ. Pollut..

[35] National Meteorological Center Blue Alert for Cold Wave. https://baijiahao.baidu.com/s?id=1687993245373928406&wfr=spider&for=pc.

[36] Jiang D., Xiao W., Wang J., Wang H., Zhao Y., Li B., Zhou P. (2018). Evaluation of the effects of one cold wave on heating energy consumption in different regions of northern China. Energy.

[37] Yan L., Li J., Liu M., Hu M., Xu Z., Xue K. (2021). Heating behavior using household air-conditioners during the COVID-19 lockdown in Wuhan: An exploratory and comparative study. Build. Environ..

[38] Pei Z., Han G., Ma X., Su H., Gong W. (2020). Response of major air pollutants to COVID-19 lockdowns in China. Sci. Total Environ..

[39] Tian X., An C., Chen Z., Tian Z. (2021). Assessing the impact of COVID-19 pandemic on urban transportation and air quality in Canada. Sci. Total Environ..

[40] Rodríguez-Urrego D., Rodríguez-Urrego L. (2020). Air quality during the COVID-19: PM(2.5) analysis in the 50 most polluted capital cities in the world. Environ. Pollut..

[41] Bherwani H., Nair M., Musugu K., Gautam S., Gupta A., Kapley A., Kumar R. (2020). Valuation of air pollution externalities: Comparative assessment of economic damage and emission reduction under COVID-19 lockdown. Air Qual. Atmos. Health.

[42] Lv Z., Wang X., Deng F., Ying Q., Archibald A.T., Jones R.L., Ding Y., Cheng Y., Fu M., Liu Y. (2020). Source-Receptor Relationship Revealed by the Halted Traffic and Aggravated Haze in Beijing during the COVID-19 Lockdown. Environ. Sci. Technol..

[43] Huang J., Pan X., Guo X., Li G. (2018). Health impact of China’s Air Pollution Prevention and Control Action Plan: An analysis of national air quality monitoring and mortality data. Lancet Planet. Health.

[44] Kunzmann K.R. (2020). Smart Cities After COVID-19: Ten Narratives. disP-Plan. Rev..

