# Supplementary material for: The Improvement of Air Quality and Associated Mortality during the COVID-19 Lockdown in One Megacity of China: An Empirical Strategy
Source: Int J Environ Res Public Health. 2021 Aug 18;18(16):8702. doi: 10.3390/ijerph18168702 (PMC8391611; doi:10.3390/ijerph18168702)
Supplement: Supplementary file 1 [file ijerph-18-08702-s001.zip › ijerph-1275889-supplementary.pdf]

Supplementary Material

Title: The improvement of air quality and associated mortality during the COVID-19 lockdown in one megacity of China: an empirical strategy

Table S1. Number of deaths due to NO_2 change during the lockdown based two references.

NO_2 (per 10 ug/m3)	Li et al, 2021 [1]	Total Deaths (17 days)	Chen et al, 2018 [2]	Total Deaths (17 days)
All-cause mortality	0.65% (0.50%, 0.80%)	14.63(11.25,18.00)	0.9% (0.7%, 1.1%)	20.25(15.75, 24.76)
CVD mortality	0.60% (0.41%, 0.79%)	5.93(4.05, 7.80)	0.9% (0.7%, 1.2%)	8.89(6.92,11.85)
Respiratory mortality	0.73% (0.46%, 1.00%)	2.00(1.26,2.74)	1.2% (0.9%, 1.5%)	3.28(2.46, 4.10)

Reference:

1. Li, J.; Zhang, X.; Li, G.; Wang, L.; Yin, P.; Zhou, M., Short-term effects of ambient nitrogen dioxide on years of life lost in 48 major Chinese cities, 2013-2017. Chemosphere 2021, 263, 127887.
2. Renjie; Chen; Peng; Yin; Xia; Meng; Lijun; Wang; Cong; Liu, Associations between ambient nitrogen dioxide and daily cause-specific mortality: Evidence from 272 Chinese cities. Epidemiology (Cambridge, Mass.) 2018.
